# The influence of post‐curing protocols on the optical properties of provisional and long‐term 3D‐printable resin composites

**DOI:** 10.1111/eos.70106

**Published:** 2026-05-14

**Authors:** Ido Luiz de Azevedo Feiten, Bruno Borges de Oliveira, Karin de Mello Weig, Luise Gomes da Motta, Luis Felipe J. Schneider, Larissa Maria Assad Cavalcante

**Affiliations:** ^1^ Laboratory for Applied Biotechnology (LABA), School of Dentistry Federal Fluminense University Niteroi Brazil; ^2^ Astro Science Uberaba Brazil

**Keywords:** cosmetic dentistry, dental materials, photoinitiators, prosthesis coloring

## Abstract

This study determined the influence of post‐curing protocols on the color stability of provisional and long‐term 3D‐printable resin composites. Disc‐shaped specimens were fabricated from a provisional (Prov)  and long‐term (Crown) 3D‐printable resin composites using a digital light processing printer. Post‐curing protocols included exposure to 405 nm‐based light sources (Anycubic for 30 or 60 min in the standard mode or Procure 1) and one 385 nm‐based light source (Procure 2 with distinct protocols). Color parameters (L*, a*, b*) were measured at baseline, after 14 and 21 d of dry storage, and after 7 d of water immersion. Post‐curing protocol, material, and storage condition significantly influenced color stability. High‐irradiance protocols resulted in the highest color change values, exceeding the clinical acceptability threshold (Δ*E*_00_ > 1.8). Water immersion induced a significant whitening effect and increased total color change in all groups. The long‐term resin demonstrated superior color stability compared to the provisional one across all post‐curing protocols. The choice of post‐curing protocol is critical for aesthetic performance, as high‐irradiance parameters, while potentially beneficial for mechanical properties, can compromise chromatic stability, particularly in materials with low filler content.

## INTRODUCTION

The integration of additive manufacturing (AM) into digital dentistry has significantly transformed clinical workflows by offering cost‐effectiveness, high material efficiency, and the capability to produce complex anatomical geometries [1]. Although subtractive milling remains a prevalent standard, 3D‐printing technologies, such as digital light processing (DLP), are increasingly adopted for fabricating both provisional and definitive restorations [2, 3]. This shift is further supported by the competitive efficiency of 3D‐printable resin composites, which offer distinct advantages in terms of production cost and time when compared to traditional subtractive materials [4, 5]. However, the success of these restorations is heavily dependent on the many steps [6, 7, 8, 9].

A critical phase in the 3D‐printing workflow is the post‐curing process, where printed objects are exposed to specific light wavelengths to maximize the degree of conversion (DC) and optimize mechanical properties [9, 10, 11]. Modern post‐curing units offer controlled parameters, including irradiance, wavelength, and temperature, which directly influence the final polymer network and material characteristics [12, 13]. The effectiveness of this stage often depends on the specific resin type and printer technology utilized [14]. Furthermore, optimizing the concentration of photoinitiators in these resins is essential for ensuring adequate polymerization for provisional applications [15]. Determining the optimal protocol that balances maximum mechanical performance with aesthetic stability remains a significant challenge for clinicians [12].

Color stability is a crucial requirement for aesthetic success but is often compromised by various intrinsic and extrinsic factors [16, 17]. In 3D‐printed resins, color changes frequently stem from the photodegradation of residual photoinitiators like diphenyl(2,4,6‐trimethylbenzoyl)phosphine oxide (TPO), as well as water sorption and the leaching of unreacted monomers [18]. Paradoxically, high‐irradiance light and elevated temperatures during post‐curing may induce oxidative reactions, resulting in early yellowing [19, 20]. The resin's composition, particularly its inorganic filler content, plays a decisive role in resisting these chromatics [19, 21]. Permanent and long‐term crown resins typically feature a higher filler load, which reduces the volume of the polymer matrix susceptible to water sorption and potentially enhances color stability compared to less‐filled provisional materials [19, 21]. Recent studies suggest that aggressive post‐curing protocols may create a trade‐off between achieving superior hardness and maintaining initial color fidelity [22, 23, 24].

Due to the limited understanding of how modern, high‐irradiance post‐curing protocols affect the color stability of different resin classes, this study aimed to evaluate these effects on a provisional and a long‐term 3D‐printed composite [25]. The tested null hypotheses were that (1) post‐curing protocols do not produce acceptability color changes (Δ*E*_00_ < 1.8) in the tested resins [26, 27, 28]; and (2) there is no difference in color stability between the provisional and the long‐term resins [25].

## MATERIAL AND METHODS

### Specimen preparation

**FIGURE 1:**
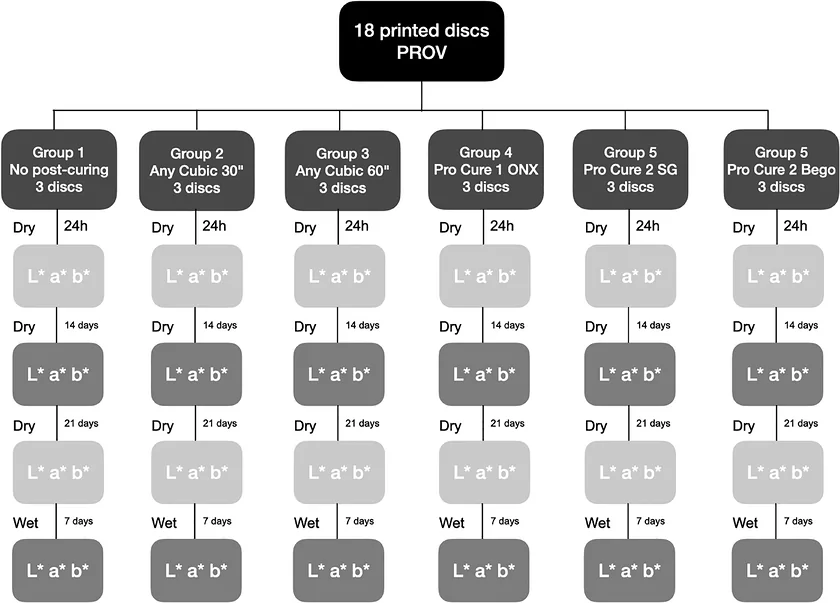
Flowchart of the steps involved in the present study Prov.

**FIGURE 2:**
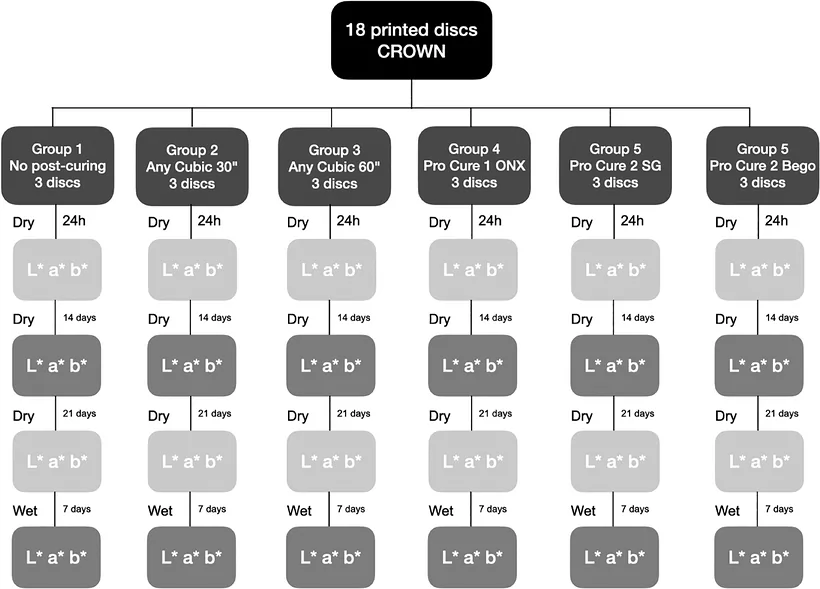
Flowchart of the steps involved in the present study Crown.

The study design is illustrated in Figures 1 and 2. Thirty‐six disc‐shaped specimens (10 mm diameter × 1 mm thickness) were digitally designed (Meshmixer, Autodesk) and 3D printed using a DLP printer (Flashforge Hunter, Zhejiang Flashforge 3D Technology). Two commercially available resins were used: a material for provisional restorations (Horus Bioprint Prov, Astro Science do Brasil) and a material for long‐term crowns (Horus Bioprint Crown, Astro Science do Brasil). These materials differ in their compositional profiles, particularly in terms of inorganic filler content and organic matrix composition, which are optimized according to their intended clinical application. The detailed chemical composition of each material is presented in Table 1. All printing parameters were set according to the manufacturers’ recommendations.

**TABLE 1:** Composition of the evaluated 3D‐printable resins according to the manufacturer's technical data (ANVISA notifications no. 10331439008 and 10331439010).

Product	Component	Content (wt%)	Manufacturer
**Horus Bioprint Prov**	Acrylic monomers and oligomers	>80	Astro Science do Brasil S.A.
	Silanized ceramic fillers	≤20	
	Photoinitiators	<2	
	Pigments	≤1	
	Photoinhibitors	<1	
**Horus Bioprint Crown**	Acrylic monomers and oligomers	>60	Astro Science do Brasil S.A.
	Silanized ceramic fillers	≤40	
	Photoinitiators	<2	
	Pigments	≤1	
	Photoinhibitors	<1	

After printing, specimens were gently removed from the build platform and underwent a standardized washing process in 99% isopropyl alcohol for 5 min, followed by air drying. The specimens of provisional and long‐term crown were divided (*n* = 3) into four post‐curing protocols, including exposure to 405 nm light (Wash & Cure Plus, Anycubic) for 30 or 60 min or using specific units (procure 1 ONX‐mode, SprintRay) and to 385 nm light (procure 2, SprintRay) in “Surgery Guide” and “Bego” modes. The control group consisted of non‐post‐cured samples.

The emission wavelengths of the post‐curing units were obtained from the manufacturers’ technical specifications. The irradiance of each unit was measured using a light meter (LS125, Linshang Technology) equipped with a UVA/UVV LED probe (UVALED‐X3, Linshang Technology). The probe has a spectral response range of 340–420 nm (calibrated at 395 nm) and a measurement range of 0–200,000 *µ*W/cm^2^. Based on these measurements, the irradiance values were 2.22 mW/cm^2^ for the Wash & Cure Plus, 5.22 mW/cm^2^ for procure 1, and 86.02 mW/cm^2^ for procure 2.

### Color stability assessment

Color parameters (L*, a*, b*) were measured using a benchtop spectrophotometer (CM‐2600d, Konica Minolta Sensing) against a white background. Measurements were taken at four time points: baseline (*T*0), 24 h after post‐curing; after 14 d of dry, dark storage at room temperature (*T*1); after 21 d of dry, dark storage (*T*2); and after an additional 7 d of immersion in distilled water following *T*2 (*T*3).

The total color difference (Δ*E*_00_) was calculated between *T*0 and each subsequent time point (*T*1, *T*2, *T*3) using the CIEDE2000 formula, with parametric factors set to 1:

(1)

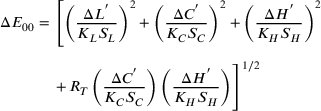

where Δ*L*′, Δ*C*′, and Δ*H*′ correspond to the mathematical differences in lightness, chroma, and hue between two moments, *R_T_* represents the rotation function; *S_L_*, *S_C_*, and *S_H_* are the weighting functions for the lightness, chroma, and hue components, and the parametric factors *K_L_*, *K_C_*, and *K_H_* are the terms to be adjusted for the evaluated conditions (defined for this study as 1). The sample size (*n* = 3) was determined based on parameters from a pilot study using the website http://estatistica.bauru.usp.br/calculoamostral [29]. The analysis considered 10 groups, *α* = 0.05, and *β* = 95%. Statistical power calculations indicated that at least 3 specimens were required for the tests.

### Statistical analysis

Statistical analysis was performed using jamovi software (Version 2.3, The Jamovi Project). Data normality and homoscedasticity were confirmed with the Shapiro–Wilk and Levene's tests, respectively. A three‐way repeated‐measures analysis of variance (anova) was used to analyze the effects of the independent variables (post‐curing protocol, material, and time) on the dependent variable (Δ*E*_00_). Tukey's HSD post hoc test was employed for pairwise comparisons. The significance level was set at *α* = 0.05.

### Artificial intelligence—assisted tools

Artificial intelligence–generated content tools (Gemini, Google) were used exclusively to assist in improving the graphical resolution of figures and in language editing for clarity and fluency. No artificial intelligence tools were used in data generation, statistical analysis, or scientific interpretation. All data analyses, results, and conclusions were performed and verified by the authors, who take full responsibility for the content of the manuscript.

## RESULTS

The three‐way repeated‐measures anova demonstrated that the post‐curing protocol, material type, and storage conditions each had a significant individual influence on the color stability of the tested resins (*p* < 0.001). Furthermore, all two‐way and three‐way interactions between these independent variables were found to be statistically significant (*p* < 0.001).

### Analysis of colorimetric coordinates (*L* ^∗^, *a* ^∗^, *b* ^∗^)

The evaluation of individual color coordinates revealed distinct patterns of chromatic evolution over the four time points. Lightness (*L* ^∗^) values increased substantially for both materials after 7 d of water immersion (*T*3), indicating a whitening effect when exposed to a moist environment.

**FIGURE 3:**
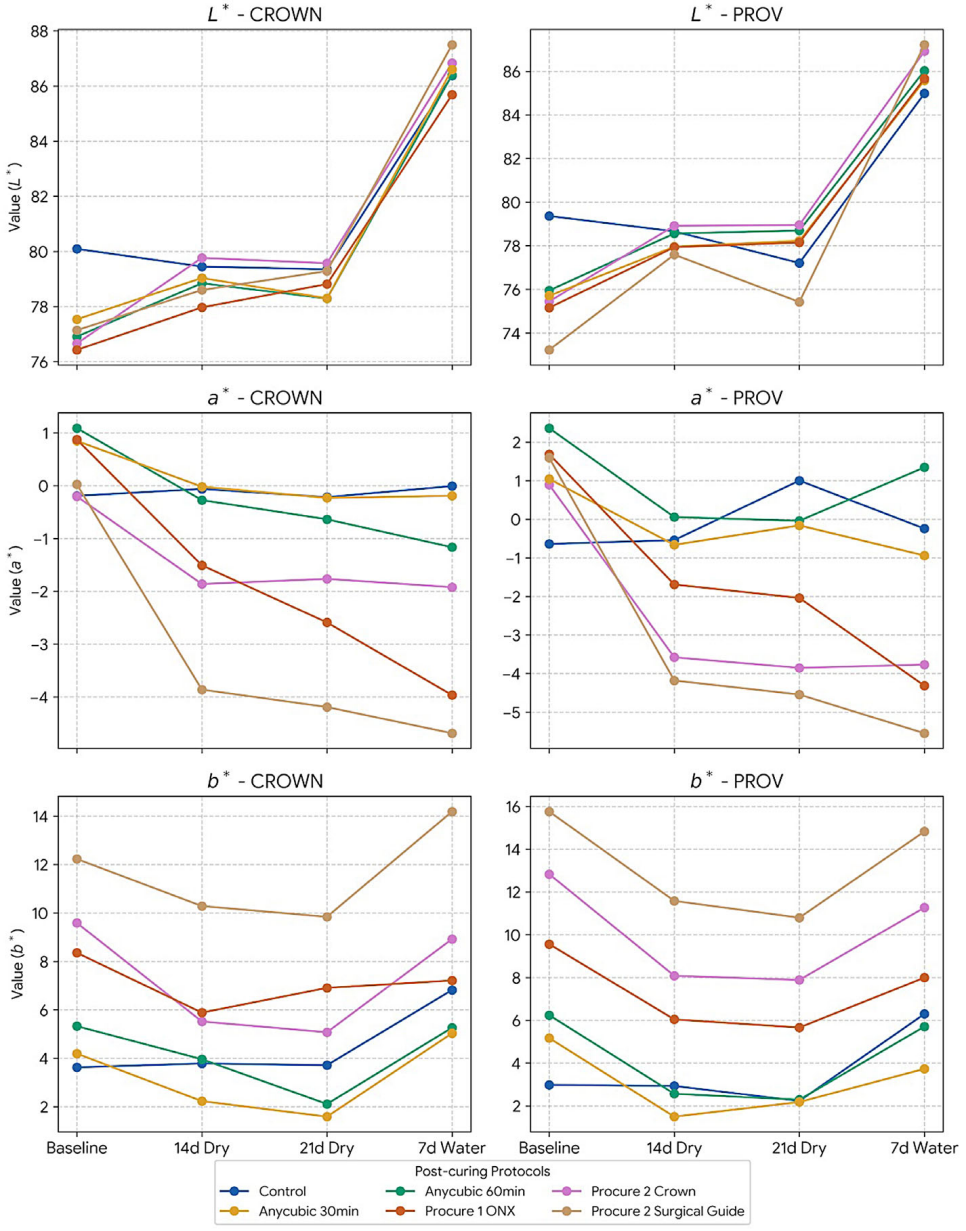
Evolution of L*, a*, b* coordinates for horus Bioprint Crown and Bioprint Prov resins across the evaluation periods: Baseline (*T*0), 14 d dry (*T*1), 21 d dry (*T*2), and 7 d in water (*T*3).

Regarding the *a* ^∗^ coordinate (green–red axis), there was a general shift toward the green spectrum (more negative values) across all groups, which was particularly pronounced in the high‐irradiance procure 1 ONX and procure 2 Surgical Guide protocols. The *b* ^∗^ coordinate was significantly influenced by the high‐performance protocols. Specimens post‐cured in the procure 2 unit (Crown and Surgical Guide modes) exhibited higher initial *b* ^∗^ values at baseline (*T*0), indicating immediate yellowing after processing. These values showed further instability following water immersion (Figure 3).

### Total color change (Δ*E*_00_) for the long‐term 3D‐printable resin composite

For the long‐term 3D‐printable resin composite, the Control group (no post‐curing) exhibited the lowest Δ*E*_00_ values during dry storage (*T*1 and *T*2), remaining below the acceptability threshold (Δ*E*_00_ < 1.8). However, the application of any post‐curing protocol resulted in significantly higher color change values. High‐irradiance protocols, specifically procure 1 ONX and procure 2 SG, consistently yielded the highest Δ*E*_00_ values. Upon water immersion (*T*3), all post‐cured groups demonstrated a significant increase in color variation. Values for procure 1 ONX (9.25) and procure 2 SG (9.12) far exceeded the clinical acceptability threshold of 1.8 (Table 2).

**TABLE 2:** Mean and standard deviation of the total color change (Δ*E*_00_) for the long‐term crown 3D‐printable resin across different post‐curing protocols and storage conditions.

Post curing	14 d dry	21 d dry	7 d wet
**Control**	0.57 (0.25) Ca	0.55 (0.23) Ca	4.95 (0.21) Ba
**Anycubic 30′**	2.42 (1.12) BCa	2.98 (1.18) BCa	6.47 (0.85) Ba
**Anycubic 60′**	3.04 (1.01) BCa	3.87 (0.30) BCa	7.22 (0.59) Ba
**procure 1 ONX**	4.08 (0.29) ABa	5.31 (0.29) Aa	9.25 (1.46) Aa
**procure 2 Bego**	4.60 (0.11) ABa	4.82 (0.19) Aa	7.36 (0.23) Ba
**procure 2 SG**	5.53 (0.18) Aa	6.12 (0.19) Aa	9.12 (0.78) Aa

### Total color change (Δ*E*_00_) for the provisional 3D‐printable resin composite

The provisional 3D‐printable resin composite followed a similar trend but displayed markedly higher Δ*E*_00_ values compared to the long‐term resin under all identical conditions. At 14 and 21 d of dry storage, the Control group was the only protocol that remained significantly more stable than the others. All post‐curing protocols resulted in high discoloration levels from the earliest measurement. Water immersion (*T*3) served as a critical accelerator for color instability, leading to the highest observed values in the study. The procure 2 surgical guide protocol reached a maximum Δ*E*_00_ of 12.90 after immersion (Table 3).

**TABLE 3:** Mean and standard deviation of the total color change (Δ*E*_00_) for the provisional 3D‐printable resin across different post‐curing protocols and storage conditions.

Post curing	14 d dry	21 d dry	7 d wet
**Control**	0.82 (0.11) Ca	3.36 (3.86) Ca	4.80 (1.30) Cb
**Anycubic 30″**	4.38 (0.92) BCa	3.71 (1.12) Ca	7.49 (0.80) BCb
**Anycubic 60″**	4.74 (0.29) BCa	5.09 (0.75) BCa	7.14 (0.84) BCb
**PC1 ONX**	5.72 (0.13) Ba	6.33 (0.12) Ba	10.92 (0.57) ABb
**PC2 Bego**	7.37 (0.12) ABa	7.80 (0.23) ABa	9.97 (0.73) Aa
**PC2 SG**	8.52 (1.34) Aa	9.34 (0.99) Aa	12.90 (0.88) Aa

## DISCUSSION

Post‐curing conditions played a critical role in the color stability of 3D‐printed resins, with distinct responses observed between provisional and long‐term materials, rejecting both null hypotheses. Although post‐polymerization is essential to enhance the DC and mechanical integrity of vat‐polymerized polymers, the protocols evaluated in this study resulted in Δ*E*_00_ values above the acceptability threshold [9, 20, 23]. These findings highlight a potential trade‐off between improved polymerization and aesthetic performance. Furthermore, whereas previous studies have suggested that extreme irradiance levels (up to 2000 W/m^2^) are required to induce rapid chemical degradation, the present results indicate that lower irradiance levels, particularly when combined with elevated temperatures (80°C), may also negatively affect chromatic stability [30].

The influence of light wavelength and irradiance during post‐curing is a primary factor in these alterations, as the reactivity of photoinitiators like TPO is highly wavelength‐dependent [31]. The procure 2 unit utilized in this study employs a 385 nm wavelength, which aligns more effectively with the absorption peak of TPO than traditional 405 nm units [31]. However, recent evidence suggests that while 385 nm light maximizes DC, high light irradiance (up to 2000 W/m^2^) can induce rapid surface and bulk chemical changes that adversely affect aesthetic characteristics [30]. In the present study, the combination of 385 nm light and elevated temperatures (80°C) likely accelerated the formation of photo‐oxidative byproducts, leading to the immediate yellowing (*b* ^∗^ increase) recorded at baseline [20, 31].

The mechanisms underlying the specific color coordinate (*L* ^∗^, *a* ^∗^, and *b* ^∗^) provide a deeper understanding of the material's degradation. The substantial increase in lightness (*L* ^∗^) observed following water immersion (*T*3) signifies a pronounced “whitening” effect, primarily driven by water sorption and the subsequent alteration of the polymer's refractive index [32, 33]. Water molecules penetrate the cross‐linked network, acting as plasticizers and increasing light scattering at the matrix‐filler interface [12, 33]. This phenomenon is exacerbated in materials where the polymer network, despite high‐irradiance curing, remains inhomogeneous, facilitating localized zones of high sorption and potential monomer elution [13, 30, 33].

Instability in the *b* ^∗^ coordinate (blue–yellow axis) further reflects the chemical instability of the initiator system under thermal stress. Elevated temperatures during post‐processing are known to accelerate the thermal degradation of residual phosphine oxide–based photoinitiators [22, 34]. TPO concentrations typically ranging from 1% to 3% in dental formulations are essential for achieving adequate depth of cure, yet higher concentrations are inherently linked to initial yellowness due to the presence of unreacted phosphine oxide groups [15]. The concomitant reduction in *a* ^∗^ values (shift toward green) suggests that high‐energy exposure may also induce the degradation of organic pigments or cause oxidative stress within the resin matrix, as seen in complex 3D‐photoprintable formulations [34, 35].

The disparity in color stability between the long‐term and provisional materials highlights the decisive role of inorganic filler content and matrix composition [21]. The long‐termcrown resin demonstrated superior stability across all protocols, likely due to its higher filler load, which effectively reduces the total volume of the water‐absorbing resin [19, 21]. A lower resin‐to‐filler ratio minimizes the space available for water sorption and limits the potential for the leaching of leachable components, such as residual photoinitiators and monomers [33]. Conversely, provisional materials often feature a higher resin‐to‐filler ratio to facilitate printing speed, rendering them more susceptible to the plasticizing effects of water and more significant Δ*E*_00_ variations [11, 14, 25].

Although the Control group exhibited the lowest Δ*E*_00_ under dry conditions, it remains a clinically unviable option. Insufficient post‐curing results in inferior mechanical properties, higher monomer elution, and increased cytotoxicity, which pose significant risks to both the structural integrity of the restoration and the biological safety of the patient [30, 33]. Compared to subtractive materials, which are industrialized under high pressure and temperature to reach higher DOC [2, 36], 3D‐printed resins require a delicate balance of wavelength, time, and temperature [12, 30]. This ensures that the 3D‐printed material reaches its maximum mechanical potential without crossing the threshold of aesthetic failure or compromising long‐term chromatic longevity [19, 30].

It is also important to recognize that color stability is not determined solely by post‐curing. Upstream steps in the printing workflow may significantly influence the final optical outcome. For instance, the specific printing technology (e.g., DLP vs. SLA or Logicon Caries Detector) dictates light scattering during layer polymerization, thereby affecting the initial polymer network structure [14]. Furthermore, the washing protocol, particularly immersion time, agitation, and isopropyl alcohol contamination, plays a critical role in the removal of unreacted monomers. Inadequate washing may leave a reactive monomer layer that is highly susceptible to discoloration during post‐curing, whereas excessive washing may compromise the surface integrity of undercured specimens. These factors should be considered in future investigations, given their potential combined effect on the final properties of printed materials [37, 38, 39].

Future research should aim to correlate specific photoinitiator substituent effects with color stability to facilitate the development of resin formulations that are inherently resistant to post‐processing discoloration [31]. This study's primary limitation is the exclusive focus on color stability without the concurrent measurement of mechanical properties or the DC. The absence of these parameters prevents a holistic view of the performance trade‐offs inherent in 3D‐printing workflows [37]. Additionally, although the in vitro testing protocol employed herein represents a highly demanding challenge to the materials’ optical stability, future studies should evaluate conditions that more closely mimic the dynamic oral environment, such as thermocycling and prolonged exposure to common staining agents (e.g., coffee, wine, or tea) [39, 40, 41, 42]. Finally, considering that the inclusion of a relatively moderate inorganic filler content (≤40 wt%) in the long‐term resin already yielded a substantial improvement in performance, future investigations should explore novel 3D‐printable formulations with even higher filler loads. Analyzing the specific particle distribution of these highly filled materials will ultimately guide the design of more stable and durable AM composites [14].

In conclusion, post‐curing parameters significantly dictate the color stability of 3D‐printed resin composites. High‐irradiance protocols utilizing elevated temperatures (e.g., 80°C) and specific wavelengths (385 nm) induce immediate chromatic shifts (yellowing) and result in Δ*E*_00_ values that far exceed the clinical acceptability threshold (Δ*E*_00_ > 1.8), particularly after exposure to water immersion. The chemical composition and filler load of the resin are decisive factors in aesthetic longevity; the long‐term crown resin demonstrated significantly superior color stability compared to the provisional material, likely due to its higher inorganic filler content and a more robust polymer matrix that limits water sorption. Consequently, clinicians and dental laboratories must adopt a calibrated approach when selecting post‐curing parameters, balancing the requirement for a high DC with the imperative of long‐term aesthetic stability. The intended clinical application (provisional vs. long‐term) must be the primary guide when determining the irradiance and duration of post‐polymerization.

## AUTHOR CONTRIBUTIONS

**Conceptualization**: Ido Luiz de Azevedo Feiten and Luis Felipe Schneider. **Formal analysis**: Karin de Mello Weig. **Investigation**: Ido Luiz de Azevedo Feiten, Luise Gomes da Motta, and Bruno Borges de Oliveira. **Methodology**: Luis Felipe Schneider and Larissa Maria Assad Cavalcante. **Writing—original draft**: Ido Luiz de Azevedo Feiten. **Writing—review and editing**: Luis Felipe Schneider and Larissa Maria Assad Cavalcante.

## CONFLICT OF INTEREST STATEMENT

Bruno Borges de Oliveira works for Astro Science do Brasil S. A. The other authors declare no conflicts of interest.
